# The impact of age and nodal status on variations in oncotype DX testing and adjuvant treatment

**DOI:** 10.1038/s41523-022-00394-1

**Published:** 2022-03-01

**Authors:** Kathleen Iles, Mya L. Roberson, Philip Spanheimer, Kristalyn Gallagher, David W. Ollila, Paula D. Strassle, Stephanie Downs-Canner

**Affiliations:** 1 Department of Surgery, University of North Carolina at Chapel Hill, Chapel Hill, NC USA; 2 Department of Health Policy, Vanderbilt University School of Medicine, Nashville, TN USA; 3 Vanderbilt-Ingram Cancer Center, Nashville, TN USA; 4 Department of Surgical Oncology, University of North Carolina at Chapel Hill, Chapel Hill, NC USA; 5 Division of Intramural Research, National Institute on Minority Health and Health Disparities (NIMHD), National Institutes of Health, Bethesda, MD USA

**Keywords:** Breast cancer, Chemotherapy, Targeted therapies

## Abstract

Oncotype DX (ODX) recurrence score (RS) is a validated tool to guide the use of adjuvant chemotherapy (AC) in hormone receptor+/HER2- breast cancer. In this analysis, we examine (1) characteristics associated with ODX testing and (2) the association between ODX RS and receipt of AC across age and nodal status. Women with HR+/HER2–, early-stage (T1-2, N0-1) breast cancers from 2010–2017 in the National Cancer Database were included. 530,125 met inclusion and 255,971 received ODX testing. Older women were less likely to receive testing; however, nodal positivity increased use of testing. High ODX RS was associated with increased mortality, though the association was not consistent across age and was most strongly associated with mortality among younger, node-negative women. Older women with high ODX RS, regardless of nodal status, were less likely to receive AC. Clinicians may be employing ODX RS to support treatment decisions against the receipt of AC.

## Introduction

Unprecedented advances in science and technology, such as multigene expression analysis of tumors, have rapidly changed treatment paradigms for many cancers. The oncotype DX (ODX) recurrence score (RS) (Exact Sciences., Madison, WI) is a validated prognostic and predictive tool to estimate patient risk of distant breast-cancer recurrence and guide the use of adjuvant chemotherapy in hormone receptor-positive (HR+) human epidermal growth factor receptor-2 negative (HER2–), early-stage breast cancer^1,2^. While the first results of RxPONDER prospective trial (NCT01272037) assessing ODX RS use in node-positive (N1) disease were reported in December 2020, a small prospective study^3^ and multiple retrospective studies have reinforced its utility in this setting^4–10^. Many clinicians have already been using the test in this cohort to guide decisions on adjuvant therapy^11^. ODX RS enables personalized treatment approaches and has reduced the use of chemotherapy, which is associated with both negative short- and long-term sequela that can severely affect quality of life^12,13^.

Cancer treatment for the aging population is challenging, requiring clinicians to critically weigh the delicate balance of competing morbidities and quality of life. Prospective randomized control trials have shown that axillary staging and radiation therapy do not improve overall or disease-specific survival in select older women with early-stage breast cancer^14–18^. Tumor biology of older breast-cancer patients is distinct, and de-implementation of practices such as adjuvant radiation and sentinel node biopsy in low-risk patients has been slow^19–22^. The National Comprehensive Cancer Network and American Society of Clinical Oncology both recommend the use of oncotype DX RS testing in HR+/HER2–, lymph node-negative or micro-metastatic tumors, however, this recommendation is currently not stratified by age or menopausal status^23,24^.

The intent of this study was to (1) investigate the prevalence of oncotype DX RS testing in women diagnosed with HR+/HER2– breast cancer and its association with mortality across age groups and nodal status, and (2) examine whether testing was associated with adjuvant chemotherapy use across both age and nodal status. We hypothesized that the predictive value of ODX RS may vary with age and nodal status and that testing and its impact on adjuvant therapy decisions may not be consistent across age groups.

## Results

Overall, 530,125 women were diagnosed with early-stage, HR+/HER2– breast cancer were included and 255,971 (48%) of these women received an ODX RS test.

### Oncotype DX recurrence score (ODX RS) testing

Demographic and clinical characteristics, stratified by testing status, are reported in Table 1. Women who received an oncotype DX RS test were on average younger, had fewer comorbidities, and lower pathologic T and N stages. Of those women who received an ODX RS test, the majority had private insurance (60% versus 40%). Most women (57%) in the untested cohort had Medicare/Medicaid. In the cohort that received an ODX RS test, node-positive women represented a smaller percentage of the total cohort compared to in the untested cohort (16% versus 20%).

**Table 1:** Patient demographics and clinical characteristics, stratified by oncotype DX recurrence score (ODX RS) testing, among women with HR+/HER2–, early-stage breast-cancer diagnosed between 2010 and 2017 in the National Cancer Database.

	Oncotype tested *N* (%)	Not tested *N* (%)	PR (95% CI)^a^
Overall^b^	255,971 (48)	274,154 (52)	–
Age group			
<40	9409 (4)	7523 (3)	1.0 (ref.)
40–69	201,869 (79)	154,213 (56)	1.01 (0.99, 1.02)
70+	44,693 (17)	112,418 (41)	0.57 (0.56, 0.58)
Race/ethnicity			
Non-Hispanic White	207,373 (83)	220,982 (83)	1.0 (ref.)
Non-Hispanic Black	19,198 (8)	22,626 (9)	0.94 (0.93, 0.95)
Hispanic	11,073 (4)	12,248 (5)	0.94 (0.93, 0.96)
Non-Hispanic Other	10,622 (4)	10,110 (4)	0.99 (0.98, 1.00)
Primary Insurance			
Private insurance	152,357 (60)	114,021 (42)	1.0 (ref.)
Medicare/Medicaid	100,561 (39)	154,206 (57)	0.89 (0.88, 0.90)
Uninsured	3053 (1)	3300 (1)	0.87 (0.84, 0.89)
Charlson-Deyo Score			
0	216,265 (84)	220,764 (81)	1.0 (ref.)
1	31,888 (12)	40,580 (15)	0.97 (0.96, 0.98)
2	5857 (2)	9112 (3)	0.92 (0.90, 0.94)
3+	1961 (1)	3698 (1)	0.85 (0.82, 0.88)
Histologic type			
Carcinoma of no special type (formerly ductal)	189,971 (74)	199,868 (73)	1.0 (ref.)
Lobular	57,941 (23)	57,964 (21)	1.03 (1.02, 1.04)
Other	8059 (3)	16,322 (6)	0.71 (0.70, 0.73)
Definitive surgery			
BCT	175,456 (69)	186,689 (69)	1.0 (ref.)
Mastectomy	80,466 (32)	87,377 (25)	0.96 (0.95, 0.97)
LN Surgery			
SLNB	162,275 (76)	144,635 (69)	1.0 (ref.)
ALND	47,496 (22)	53,768 (26)	0.91 (0.90,0.92)
No surgery	3216 (2)	11,858 (6)	0.57 (0.55, 0.59)
Pathologic N stage			
0	213,433 (83)	215,334 (78)	1.0 (ref.)
I	41,636 (16)	53,587 (20)	0.83 (0.82, 0.84)
Not staged	902 (1)	5233 (2)	0.67 (0.62, 0.72)
Pathologic T stage			
I	192,958 (75)	215,032 (78)	1.0 (ref.)
II	63,013 (25)	59,122 (22)	1.17 (1.16,1.18)

*PR* prevalence ratio, *CI* confidence interval, *LN* lymph node, *SLNB* sentinel lymph node biopsy, *ALND* axillary lymph node dissection.

^a^Adjusted for all variables in the table and year of diagnosis.

^b^Rows by section for race/ethnicity, primary insurance type, definitive surgery and lymph node surgery do not sum to overall counts due to missing values for these variables.

**Fig. 1:**
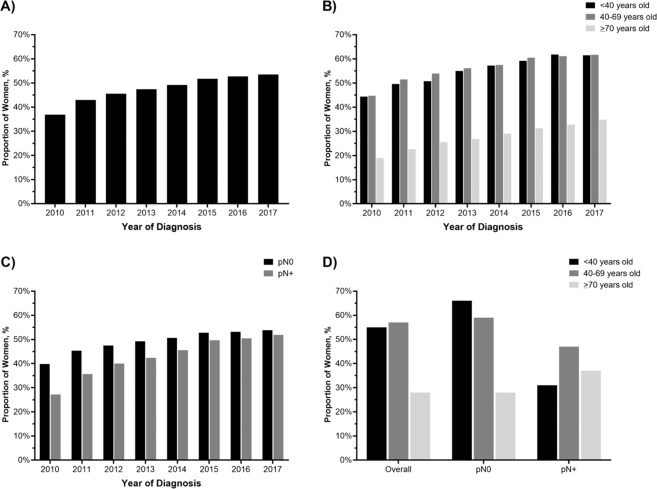
Trends in oncotype DX recurrence score (ODX RS) testing over time. **A** overall and stratified by **B** age group **C** nodal status, and **D** age and nodal status, among women with HR+/HER2–, early-stage breast cancer.

When we examined the association between pathologic nodal status and receipt of an ODX RS test across age, we found that nodal positivity was not a consistent predictor of testing, *p* < 0.0001. Among women <40 and 40–69 years old, node-positive women were less likely to undergo oncotype DX RS testing (<40: prevalence ratio [PR] 0.45, 95% CI 0.43, 0.47; 40–69: PR 0.74, 95% CI 0.73, 0.74), compared to their node-negative counterparts; however, nodal positivity was associated with increased use of ODX RS in women ≥70 (PR 1.26, 95% CI 1.23, 1.28) (Fig. 1D).

### Oncotype DX recurrence score and adjuvant chemotherapy

**Fig. 2:**
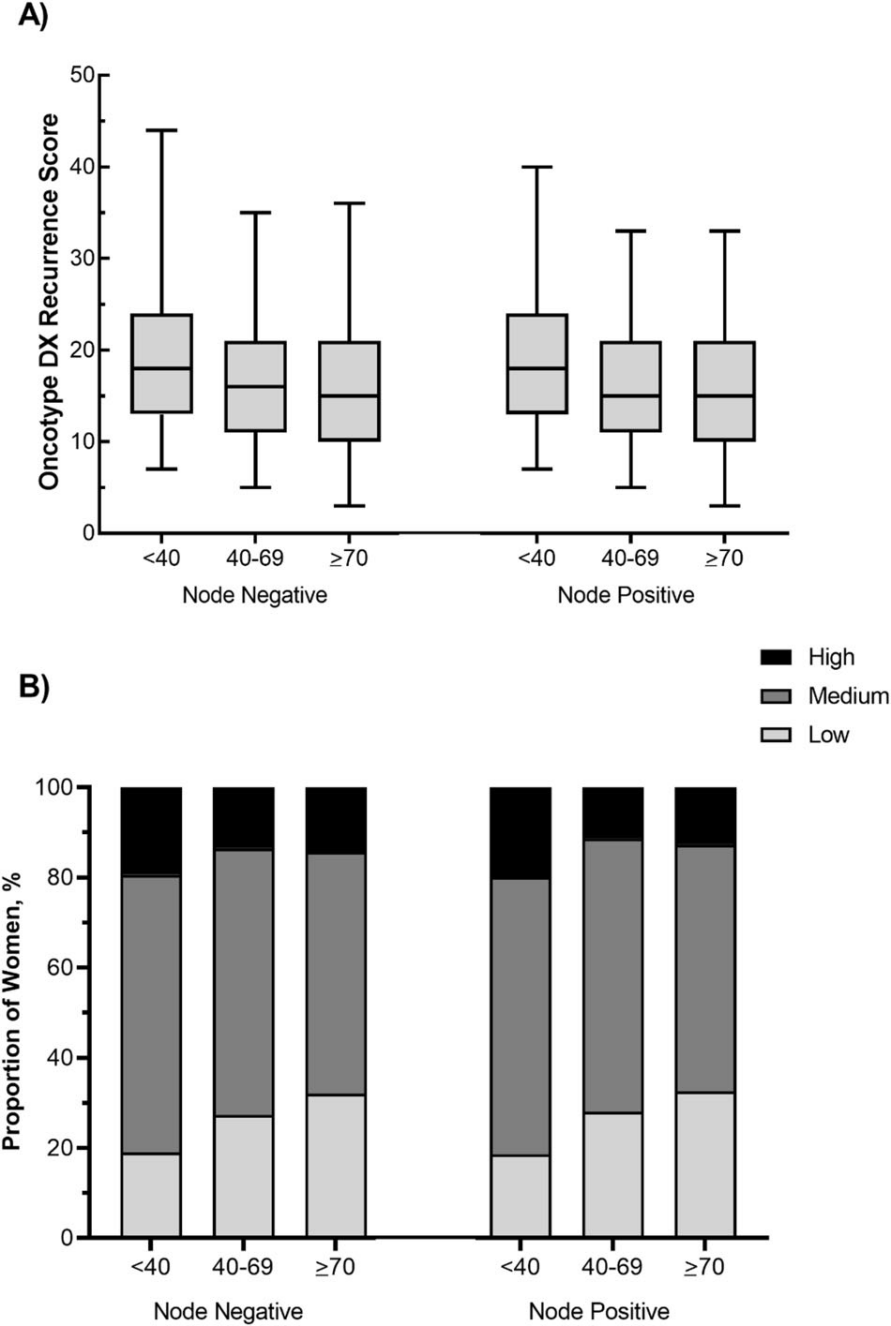
Median Oncotype DX recurrence scores stratified by age and nodal status. The **A** median oncotype DX recurrence score (ODX RS) and **B** categorized ODX RS, stratified by age group and nodal status among women with HR+/HER2–, early-stage breast cancer.

Of the women who had oncotype DX RS testing (*n* = 255,971), 248,576 women had complete information including numerical ODX RS. Median oncotype score was similar across age groups and nodal positivity status, with a median score ranging from 15–18 (Fig. 2A). Distribution of high, intermediate and low ODX recurrence scores across age subgroups and nodal status are depicted in Fig. 2B. High ODX RS was most prevalent in women <40 and low ODX RS was most prevalent in women ≥70, regardless of nodal status.

Receipt of chemotherapy, stratified by age cohort and oncotype DX RS, is shown in Table 2. After adjustment for Charlson score, insurance status, tumor grade, histologic subtype and tumor stage, ODX RS had the smallest impact on adjuvant chemotherapy use among younger women (<40 years old), with high ODX RS, compared to low ODX RS, associated with a fourfold increase in receipt of adjuvant chemotherapy. Among women 40–69 years old and ≥70 years old, high ODX RS, compared to low, was associated with substantially higher prevalence of adjuvant chemotherapy use (40–69: 16.10, 95% CI 15.4, 16.83; ≥70: 30.85, 95% CI 26.73, 35.61).

**Table 2:** Prevalence of adjuvant chemotherapy initiation within 9 months of definitive diagnosis, stratified by Oncotype DX recurrence score (ODX RS) and nodal status, among women with HR+/HER2–, early-stage breast cancer who underwent ODX RS testing, *n* = 248,576.

	<40 years old		40–69 years old		≥70 years old	
	*N* (%)	PR (95% CI)^a^	*N* (%)	PR (95% CI)^a^	*N* (%)	PR (95% CI)^a^
Overall	9144 (3.6)	–	196,271 (79.0)	–	43,161 (17.4)	–
ODX RS						
Low (≤10)	1737 (19.0)	1.0 (ref.)	53, 922 (27.5)	1.0 (ref.)	13,829 (32.0)	1.0 (ref.)
Intermediate (11–25)	5623 (61.5)	2.48 (2.19, 2.81)	116,613 (59.4)	4.15 (3.97, 4.34)	23,291 (54.0)	4.02 (3.46, 4.67)
High (≥26)	1784 (19.5)	4.94 (4.37, 5.57)	25,736 (13.1)	16.10 (15.40, 16.83)	6041 (14.0)	30.85 (26.73, 35.61)
Nodal status^b^						
pN0	7630 (83.6)	1.0 (ref.)	164,981 (84.3)	1.0 (ref.)	34,643 (80.7)	1.0 (ref.)
pN1	1496 (16.4)	1.13 (1.10, 1.16)	30,711 (15.7)	1.46 (1.43, 1.48)	8254 (19.2)	1.35 (1.27, 1.43)

*PR* prevalence ratio, *CI* confidence interval, *ODX RS* oncotype DX recurrence score.

^a^Adjusted for age group, oncotype DX recurrence score, Charlson score, insurance status, grade, cancer type, pathologic T stage; interaction terms between age and ODX RS and nodal status, respectively, were used to get age-stratified effect estimates.

^b^Patients who had missing staging information were excluded from analysis.

Sub-analysis was performed on receipt of adjuvant chemotherapy in women who received a high (≥26) ODX RS (Supplemental Table II). Of all women who received a high ODX RS, younger women were more likely to receive adjuvant chemotherapy than their older counterparts, (88.8% and 77.2% versus 51.3% in the <40, 40–69, and ≥70-year-old cohorts, respectively). Nodal positivity resulted in increased use of adjuvant chemotherapy across all age subgroups. However, among women over 70, adjuvant chemotherapy was used in 55% of node-positive and 51% of node-negative women with high ODX RS.

### Oncotype DX recurrence score and mortality

**Fig. 3:**
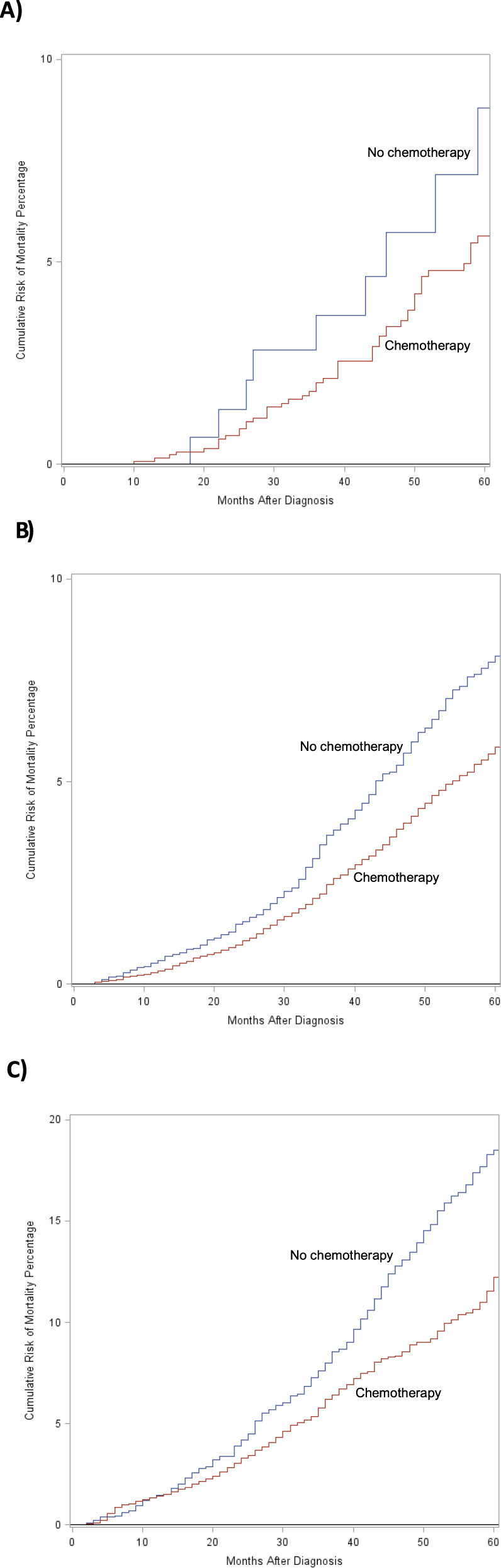
Cumulative incidence of 5-year, all-cause mortality among women with high oncotype DX recurrence score (ODX RS), stratified by age and adjuvant chemotherapy status. Blue line indicates no chemotherapy and red line indicates chemotherapy received. **A** Women <40, **B** Women 40–69, **C** Women ≥70.

**Fig. 4:**
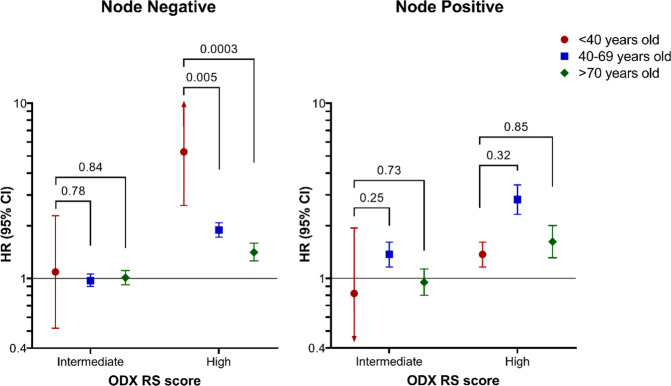
Interaction between age group and nodal positivty and its association with mortality. Interaction between age group and nodal positivity on 5-year, all-cause mortality, among women with HR+/HER2–, early-stage breast cancer who had an oncotype DX recurrence score (ODX RS) test. Models were adjusted for Charlson-Deyo score, histologic subtype, pathologic T stage, tumor grade, and insurance status.

The overall, 5-year cumulative incidence of mortality among women diagnosed between 2010 and 2017 was 9.4% (95% CI 9.3, 9.5; among women with ODX RS test: 4.6%, 95% CI 4.5, 4.7). After adjusting for Charlson score, insurance status, tumor grade, histologic subtype and tumor stage, older age and high ODX RS were all associated with increased mortality, among women who were tested, Fig. 3. Cumulative risk of mortality increased with advanced age, regardless of receipt of chemotherapy (Fig. 3). Women who received adjuvant chemotherapy had a lower cumulative mortality risk. In node-negative women, high ODX RS was strongly associated with increased mortality in younger women (<40: HR 5.28, 95% CI 2.61, 10.66), but the association significantly weakened as age increased (40–69: HR 1.89, 95% CI 1.72, 2.08; ≥70: HR 1.41, 95% CI 1.26, 1.59), (Fig. 4). In node-positive women, ODX was still associated with mortality, but was not consistent across age groups (<40: HR 1.76, 95% CI 0.72, 4.34; 40–69: HR 2.82, 95% CI 2.32, 3.43; ≥70: HR 1.62, 95% CI 1.31, 2.00), and was weaker than among young, node-negative women.

## Discussion

Our study highlights the current landscape of oncotype DX RS testing; that is, older women are less likely to receive an ODX RS test than their younger counterparts, but when RS testing is used in in women ≥70 years of age, it is more often performed in those with nodal positivity. High ODX RS was associated with higher all-cause mortality across nodal status. However, this relationship was not consistent across age and high ODX RS was most strongly associated with mortality among younger, node-negative women. Older women with high ODX RS, regardless of nodal status, were less likely to receive adjuvant chemotherapy.

Our data is consistent with previous studies^25–29^ demonstrating decreased use of ODX RS with increasing age. Similar to previous reports^30^, we found that median ODX RS was similar across age and nodal status. In a study of 610,350 tumor specimens from node-negative, micro-metastatic and node-positive patients, Bello et al. demonstrated the independent nature of tumor biology and anatomic stage by showing similar distributions of ODX scores across nodal status. A second study showed a similar distribution of ODX RS across premenopausal and post-menopausal cohorts^24^. Despite similar median scores across age groups, older, node-positive women have a higher prevalence of low oncotype scores, very low rates of high oncotype scores, and high ODX scores are more frequent in the very young (<40)^8,31^. Our data similarly demonstrated a higher prevalence of low oncotype DX RS in women ≥70 years of age despite similar median scores to younger patients.

As found in prior studies^32,33^, younger age and nodal positivity were associated with increased use of adjuvant chemotherapy. Past literature has found 70–88% of women in receipt of a high ODX RS receive adjuvant chemotherapy^32–35^. Similarly, our analysis found receipt of AC following a high (≥26) ODX recurrence score to be 88.8% and 77.2% in women ≤40 years and 40–69 years, respectively. When stratified by <50 or ≥50 years of age, Williams et al. found no difference in chemotherapy recommendation based on ODX RS^36^. However, in our cohort, among women over 70 years of age, only about half with high ODX RS (node negative or node positive) received adjuvant chemotherapy. This suggests that treatment decisions begin to differ not only with menopausal status (using age 50 as a surrogate), but instead among those with perceived competing risks for mortality. The recent presentation of the RxPONDER data confirms the safety of chemotherapy omission in post-menopausal women with 1–3 positive nodes and ODX RS <25^37^. While various reasons could explain the differential outcomes in pre- and post-menopausal women (including the ovarian suppressive effect of cytotoxic chemotherapy), these data may also reflect different tumor biology across age groups. Although our results describe the current landscape of the use and implications of ODX RS testing, further study examining the impact of these outcomes following the release of RxPONDER will be of interest.

The overall health benefit of chemotherapy must be considered in terms of decreased risk of relapse and improved survival^36,38^. Important drivers of this decision include estimation of life expectancy, chemotherapy-derived toxicity and the possibility of functional decline^39^. The decisions to recommend use ODX testing and use adjuvant chemotherapy, when indicated, are multifaceted and require insight into patient comorbidity and quality of living. Functional age, rather than chronological age, should be considered. Notably, a recent questionnaire-based prospective study studying the impact of chemotherapy on global health status in patients over 70 years of age found the negative impact of chemotherapy on quality life was statistically significant at 6 months, however, chemotherapy was no longer impactful on quality of life by 18 months^13^. Multiple validated tools have been proposed to aid physicians in objectively measuring the medical and psychosocial domains of the geriatric patient^40^. As observed in our data, oncotype DX RS testing is currently being used in the elderly breast-cancer population, however, a high ODX RS did not consistently result in receipt of chemotherapy in this cohort. Clinicians should consider comprehensive geriatric assessments before making treatment decisions so that costly procedures and tests, that may also have potential risks, can be avoided.

In our data, high ODX RS was most strongly associated with mortality among younger, node-negative women and this association weakened as age increased. The relationship between high oncotype DX RS and mortality persists across all ages and nodal status. We suspect these findings may be a result of both varied biology across age groups and a reflection of the bias of who clinicians elect to order the test for. Despite these differences, receipt of chemotherapy was associated with reduced mortality for women with high ODX RS in all age groups. Retrospective analyses have also demonstrated that the receipt of chemotherapy results in improved overall survival not only in older women, but also in women with multiple comorbidities^41,42^. However, in a Surveillance, Epidemiology and Endports Registry (SEER) study, Zhou and colleagues^43^ showed that while ODX RS was a strong predictor of breast-cancer-specific mortality, the addition of adjuvant chemotherapy did not decrease the risk of breast-cancer-specific mortality in a competing risk model. In this context, our data again highlights the importance of assessing competing risks and patient preferences when choosing to use and apply results of the ODX RS test.

Our analysis is not without limitations. The NCDB lacks cancer-specific survival data. While this has implications for the older patients in this investigation, due to their competing comorbid conditions, we believe the data is still informative in helping us understand the value of oncotype in older and node-positive women. We acknowledge that our findings may not fully describe the spectrum of ODX RS in relation to nodal status and all-cause mortality given the implications of selection bias. To better contextualize our results, we conducted analyses to understand the characteristics of who received testing compared to who did not. In spite of these limitations, we believe our analysis likely reflects real world practice of how clinicians are currently using this tool.

We have shown that oncotype DX testing is used less frequently in node-negative women over 70 years of age compared to younger women, and more commonly in node-positive women ≥70 years. We demonstrate that high ODX score is most predictive of survival in the youngest patients. Similarly, and not surprisingly, adjuvant chemotherapy in women with high ODX RS of all ages and nodal statuses, is associated with reduced mortality, however, it is given only about half the time in women ≥70 with a high ODX RS. As we have elected to omit invasive procedures and treatments in this patient population, we recommend thoughtful use of ODX RS, taking into consideration life expectancy and patient values.

## Methods

### Study design and patient population

This retrospective cohort study used data from the National Cancer Database (NCDB), a joint database project of the Commission on Cancer (CoC) and the American College of Surgeons capturing incident cancer cases. Established in 1989, this nationwide, facility-based registry captures ~70% of newly diagnosed cancer cases each year. This registry captures valuable patient and cancer characteristics in addition to therapeutics avenues in a de-identified fashion. Institutional Review Board approval was not required as the NCDB is a de‐identified, publicly available database.

**Fig. 5:**
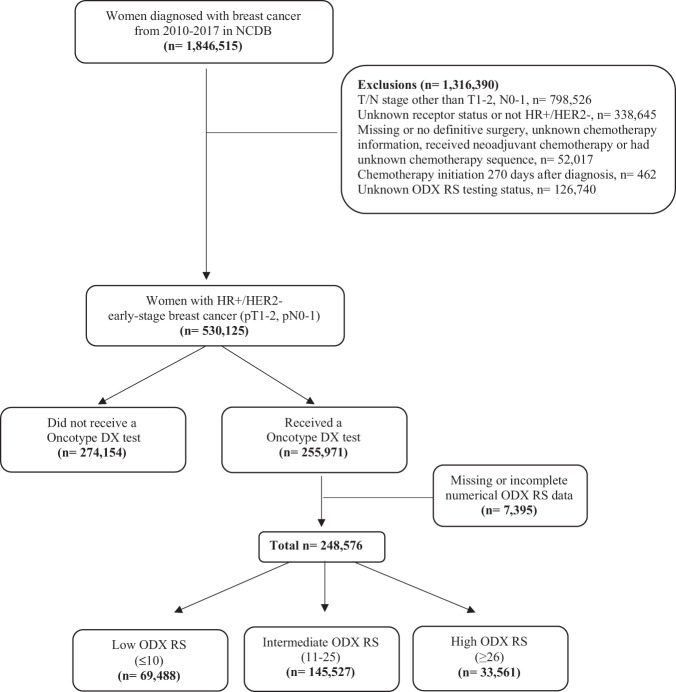
Flowchart of inclusion and exclusion criteria.

Adult women (≥18 years old) diagnosed with hormone receptor (HR)+/HER2– early-stage breast cancers (T1-2, N0-1) from 2010–2017 were identified in the NCDB, (Fig. 5). Women were excluded if they had neoadjuvant chemotherapy, had evidence of metastatic disease (cM1 or pM1), or did not undergo definitive surgery. Women were then categorized as having undergone ODX RS testing or not. Among women who were tested, scores were categorized as low (≤10), intermediate (11–25) and high (≥26) subgroups. These cutoff points are the same as used in the TailorX trial^2^.

The two primary outcomes of interest were adjuvant chemotherapy initiation within 9 months of definitive diagnosis and 5-year and all-cause mortality. Women were followed until loss-to-follow-up, death, or December 2017.

### Statistical analysis

Descriptive statistics were used to characterize pertinent demographics and clinical characteristics, including race, Charlson-Deyo comorbidity score, insurance type, residence type, residential income, clinical stage, tumor grade, histologic subtype, in addition to pathologic T and N stages.

Multivariable log-binomial regression was used to assess differences in the prevalence of ODX RS use across patient and cancer characteristics. An additional model with interaction terms was used to assess whether the association between testing and nodal status was consistent across age. Multivariable log-binomial regression was also used to assess whether ODX RS score was predictive of adjuvant chemotherapy use. Women who underwent ODX RS testing but were missing an associated numerical score were excluded from these analyses (*n* = 7395). Separate models with interaction terms between ODX RS score and age group were also run among node-negative and node-positive women. Models were adjusted for relevant covariates based on outcome of interest and are indicated accordingly within each analysis.

Kaplan-Meier curves were then used to examine survival in women with high ODX RS across age and nodal status by receipt of chemotherapy. Multivariable Cox proportional hazards regression and interaction terms were used to assess whether the association between ODX RS and age on 5-year, all-cause mortality. Separate models were again run among node-negative and node-positive women. Women diagnosed in 2017 were excluded from this analysis, as NCDB suppresses follow-up data among women diagnosed in the last year of available data (2017).

All analyses were performed using SAS version 9.4 (SAS Inc., Cary, NC). The University of North Carolina Institutional Review Board deemed this study exempt (IRB# 20–1493).

### Reporting summary

Further information on research design is available in the Nature Research Reporting Summary linked to this article.

## Supplementary information

Supplemental Materal

Reporting Summary

## Data Availability

Data accessible through National Cancer Database (NCDB) Participant User Data File (PUF) and can be accessed at https://www.facs.org/quality-programs/cancer/ncdb/puf.

## Code availability

Analytical coding will be shared upon request.
