# Supplementary material for: The impact of age and nodal status on variations in oncotype DX testing and adjuvant treatment
Source: NPJ Breast Cancer. 2022 Mar 1;8:27. doi: 10.1038/s41523-022-00394-1 (PMC8888624; doi:10.1038/s41523-022-00394-1)

Supplemental Tables and Figures

Supplemental Table I. Patient demographics and clinical characteristics associated with all-cause, 5-year mortality, among women with HR+/HER2-, early stage breast cancer diagnosed between 2010-2016.

	**HR (95% CI)**[a]	**p-value**
**Age Group**		
<40	1.0 (ref)	–
40-69	1.66 (1.32, 2.11)	<0.0001
70+	3.37 (2.65, 4.30)	<0.0001
**Race/Ethnicity**		
Non-Hispanic White	1.0 (ref)	–
Non-Hispanic Black	1.15 (1.05, 1.25)	0.0036
Hispanic	0.61 (0.51, 0.72)	<0.0001
Non-Hispanic Other	0.58 (0.48, 0.71)	<0.001
**Primary Insurance**		
Private insurance	1.0 (ref)	–
Medicare/Medicaid	2.03 (1.90, 2.18)	<0.0001
Uninsured	1.62 (1.24, 2.11)	0.0003
**Charlson-Deyo Score**		
0	1.0 (ref)	–
1	1.79 (1.68, 1.92)	<0.0001
2	2.60 (2.31, 2.92)	<0.0001
3+	3.87 (3.25, 4.61)	<0.0001
**Histologic type**		
Ductal	1.0 (ref)	–
Lobular	0.80 (0.75, 0.86)	<0.0001
Other	0.84 (0.71, 0.99)	0.0391
**Definitive Surgery**		
BCT	1.0 (ref)	–
Mastectomy	1.06 (1.0, 1.13)	0.0503
**LN Surgery**		
SLNB	1.0 (ref)	–
ALND	1.08 (1.01, 1.15)	0.0224
No surgery	1.39 (1.10, 1.75)	0.0050
**Adjuvant chemotherapy**[b]		
**Yes**	1.0 (ref)	-
**No**	0.76 (0.71, 0.81)	<0.0001
**Pathologic N stage**		
0	1.0 (ref)	–
I	1.17 (1.10, 1.26)	<0.0001
Not staged	1.35 (0.95, 1.93)	<0.0001
**Pathologic T stage**		
I	1.0 (ref)	–
II	1.66(1.57, 1.77)	<0.0001

Abbreviations: HR, hazard ratio; CI, confidence interval; LN, lymph node; SLNB, sentinel lymph node biopsy; ALND, axillary lymph node dissection

[a] Adjusted for all variables in the table and year of diagnosis

[b] Treated as a time-varying covariate; women were considered unexposed to adjuvant chemotherapy until their first treatment date

Supplemental Table II. Receipt of adjuvant chemotherapy among women diagnosed with HR+/HER2-, early-stage breast cancer from 2010-2017 and received a high (≥26) ODX RS, stratified by nodal status.

**All** Women with High ODX RS	**<40 Years N (%)** (n= 1,784)	**40-69 Years N (%)** (n=25,736)	**70+ Years N (%)** (n=6,041)
	1,584 (88.8)	19,867 (77.2)	3,099 (51.3)

**Node Negative** Women with High ODX RS	**<40 Years N (%)** (n= 1,482)	**40-69 Years N (%)** (n=22,203)	**70+ Years N (%)** (n=4,955)
	1,309 (88.3)	17,005 (76.6)	2,502 (50.5)

**Node Positive** Women with High ODX RS	**<40 Years N (%)** (n= 296)	**40-69 Years N (%)** (n=3,445)	**70+ Years N (%)** (n=1,040)
	269 (90.9)	2,803 (81.4)	576 (55.4)

Supplemental Figure I. Receipt of adjuvant chemotherapy from 2010-2017 in the National Cancer Database of women 70+ years of age with high oncotype DX Recurrence Score (ODX RS), stratified by nodal status

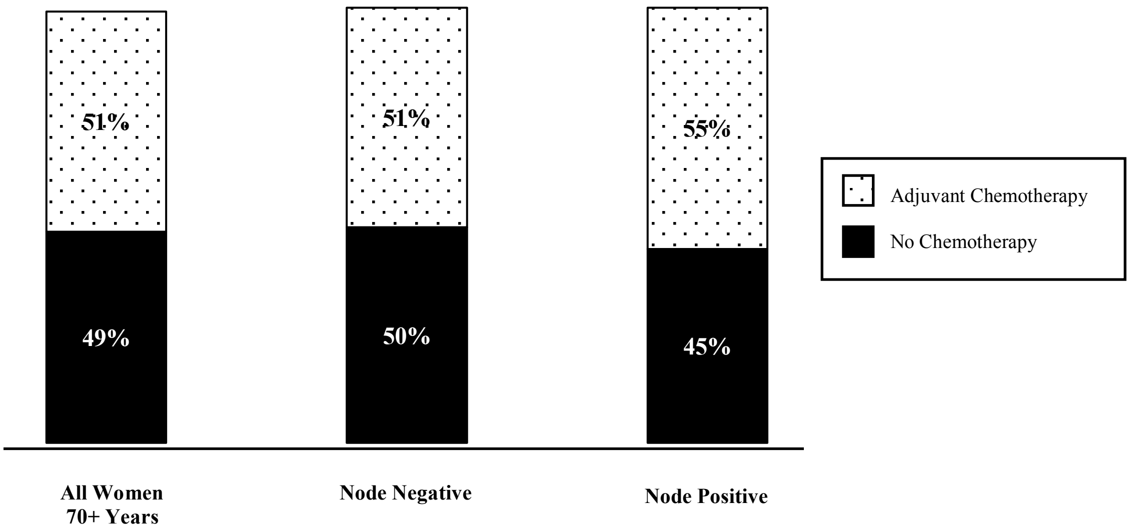

Supplement: Supplementary file 1 — Supplemental Materal [file 41523_2022_394_MOESM1_ESM.pdf]
