# Supplementary material for: The impact of age and nodal status on variations in oncotype DX testing and adjuvant treatment
Source: NPJ Breast Cancer. 2022 Mar 1;8:27. doi: 10.1038/s41523-022-00394-1 (PMC8888624; doi:10.1038/s41523-022-00394-1)
Supplement: Supplementary file 2 — Reporting Summary [file 41523_2022_394_MOESM2_ESM.pdf]

nature portfolio

Corresponding author(s): Stephanie Downs-Canner

Last updated by author(s): 2021/11/03

Reporting Summary

Nature Portfolio wishes to improve the reproducibility of the work that we publish. This form provides structure for consistency and transparency in reporting. For further information on Nature Portfolio policies, see our Editorial Policies and the Editorial Policy Checklist.

Statistics

For all statistical analyses, confirm that the following items are present in the figure legend, table legend, main text, or Methods section.

n/a	Confirmed	
☐	☒	The exact sample size (n) for each experimental group/condition, given as a discrete number and unit of measurement
☐	☒	A statement on whether measurements were taken from distinct samples or whether the same sample was measured repeatedly
☐	☒	The statistical test(s) used AND whether they are one- or two-sided<br>*Only common tests should be described solely by name; describe more complex techniques in the Methods section.*
☐	☒	A description of all covariates tested
☐	☒	A description of any assumptions or corrections, such as tests of normality and adjustment for multiple comparisons
☐	☒	A full description of the statistical parameters including central tendency (e.g. means) or other basic estimates (e.g. regression coefficient) AND variation (e.g. standard deviation) or associated estimates of uncertainty (e.g. confidence intervals)
☐	☒	For null hypothesis testing, the test statistic (e.g. F, t, r) with confidence intervals, effect sizes, degrees of freedom and P value noted<br>*Give P values as exact values whenever suitable.*
☒	☐	For Bayesian analysis, information on the choice of priors and Markov chain Monte Carlo settings
☒	☐	For hierarchical and complex designs, identification of the appropriate level for tests and full reporting of outcomes
☒	☐	Estimates of effect sizes (e.g. Cohen's d, Pearson's r), indicating how they were calculated

Our web collection on statistics for biologists contains articles on many of the points above.

Software and code

Policy information about availability of computer code

Data collection	All analyses were performed using SAS version 9.4 (SAS Inc., Cary, NC).
Data analysis	All analyses were performed using SAS version 9.4 (SAS Inc., Cary, NC). Analytical coding will be shared upon request.

For manuscripts utilizing custom algorithms or software that are central to the research but not yet described in published literature, software must be made available to editors and reviewers. We strongly encourage code deposition in a community repository (e.g. GitHub). See the Nature Portfolio guidelines for submitting code & software for further information.

Data

Policy information about availability of data

All manuscripts must include a data availability statement. This statement should provide the following information, where applicable:

- Accession codes, unique identifiers, or web links for publicly available datasets
- A description of any restrictions on data availability
- For clinical datasets or third party data, please ensure that the statement adheres to our policy

Data accessible through National Cancer Database (NCDB) Participant User Data File (PUF). Analytical coding will be shared upon request.

Field-specific reporting

Please select the one below that is the best fit for your research. If you are not sure, read the appropriate sections before making your selection.

☒ Life sciences ☐ Behavioural & social sciences ☐ Ecological, evolutionary & environmental sciences

For a reference copy of the document with all sections, see nature.com/documents/nr-reporting-summary-flat.pdf

Life sciences study design

All studies must disclose on these points even when the disclosure is negative.

Sample size	Sample size determination was made based upon availability of data in the National Cancer Database, which collects data on approximately 70% of incident cancer cases in the United States.
Data exclusions	Exclusions were pre-established based upon the specific hypothesis-directed question and to create our patient cohort from the existing data. Women were excluded if they had neoadjuvant chemotherapy, had evidence of metastatic disease (cM1 or pM1), or did not undergo definitive surgery.
Replication	Not relevant to study design
Randomization	Not relevant to study design
Blinding	Blinding was not possible as this was based on a retrospectively collected dataset

Reporting for specific materials, systems and methods

We require information from authors about some types of materials, experimental systems and methods used in many studies. Here, indicate whether each material, system or method listed is relevant to your study. If you are not sure if a list item applies to your research, read the appropriate section before selecting a response.

Materials & experimental systems

n/a	Involved in the study	
☒	☐	Antibodies
☒	☐	Eukaryotic cell lines
☒	☐	Palaeontology and archaeology
☒	☐	Animals and other organisms
☐	☒	Human research participants
☒	☐	Clinical data
☒	☐	Dual use research of concern

Methods

n/a	Involved in the study	
☒	☐	ChIP-seq
☒	☐	Flow cytometry
☒	☐	MRI-based neuroimaging

Human research participants

Policy information about studies involving human research participants

Population characteristics	Adult women (≥18 years old) diagnosed with hormone receptor (HR)+/HER2- early-stage breast cancers (T1-2, N0-1) from 2010-2017 were identified in the National Cancer Database. Adult women (≥18 years old) diagnosed with hormone receptor (HR)+/HER2- early-stage breast cancers (T1-2, N0-1) from 2010-2017 were identified in the NCDB
Recruitment	N/A , this was a retrospective study
Ethics oversight	The University of North Carolina Institutional Review Board deemed this study exempt (IRB# 20-1493).

Note that full information on the approval of the study protocol must also be provided in the manuscript.
